# Training, or Forced Exercise in the Treatment of Diabetes

**Published:** 1867-09

**Authors:** 


### TRAINING, OR FORCED EXERCISE IN THE TREATMENT OF DIABETES.

Professor Bouchardat, while admitting the efficacy of alimentary treatment in diabetes, considers it only as palliative, and he recommends the adoption of energetic exercise. This idea is not a novelty on his part, as in former writings he recommended, in the case of patients affected with this complaint, the energetic action of their bodies and arms; and then he ascertained that labor in the open air always promotes the utilization of the feculent matters in diabetic patients. It is not sufficient in all cases to cause the disappearance of the sugar, but, all things being equal, in regard to the quantity of feculent matters absorbed and other conditons, a diminution in the proportion of sugar contained in the urine always coincided with exercise in the open air. M. Bouchardat gives an instance of remarkable success in the treatment of diabetes attained by this treatment, the diet being carefully regulated and the urine being examined at intervals. Although the patient may at first be very weak, the adoption of exercise will gradually give him strength. It is of the greatest importance, according to M. Bouchardat, to use the strength in proportion as it returns; and daily exercise of the body, arms, and legs is indispensable. The greatest care must be taken to find some daily exercise which is agreeable to the patient; as, for instance, in the case of men, hunting, rowing, fencing, skating, billiards, cricket, etc., or any ordinary manual employment, as sawing, cleaving wood, turning, and the active work of gardening; and in women, all the active household employments, especially those which require the action of the legs rather than standing without walking. Riding in a carriage is not to be adopted except when no other exercise is possible; but riding on horseback is a salutary kind of movement, although it cannot be substituted for all the others. Of all the modes of exercise, that which is most convenient must be chosen; and it ought to be energetic, so as to produce a thorough sweating over the whole body; and then all necessary precaution should be taken to prevent the chance of chilling the system. M. Bouchardat relates several cases in which his system was successfully adopted in the treatment of diabetes; he considers the exercise of the gymnasium especially useful when such an establishment is well conducted, and he gives some rules to be followed by the patients. When the exercise has been continued for about an hour, and all the body is bathed in sweat, the flannel should be changed, and the skin washed briskly with cloths soaked in cold water, then strongly rubbed with coarse gloves or towels, or flesh-brushes. Then the body is to be struck and kneaded, so as to produce a complete reaction, which is sustained by a walk of a quarter of an hour at least, the body being protected by good woolen clothes. The skin should not be neglected while these exercises are used, and salt-water baths, either warm, or, what is better, cold, if they can be borne, are, according to M. Bouchardat, of almost invariable utility. During the treatment the diet must be carefully regulated, glycogenic substances being avoided while the urine is diabetic, and resumed only when the sugar has disappeared.

The red wines of Bordeaux or Burgundy may be drunk; but sparkling wines, like champagne, should be avoided. Coffee and tea, without sugar, are sometimes suitable, but their employment must be regulated by the condition of the urine after they are taken.—*British and Foreign Medico-Chirurgical Review, Oct., 1866.*

